# Can intervals enhance the inflammatory response and enjoyment in upper-body exercise?

**DOI:** 10.1007/s00421-017-3602-4

**Published:** 2017-04-04

**Authors:** Sven P. Hoekstra, Nicolette C. Bishop, Christof A. Leicht

**Affiliations:** The Peter Harrison Centre for Disability Sport, School of Sport, Exercise, and Health Sciences, Loughborough University, Towers Way, Loughborough, LE11 3TU UK

**Keywords:** Cytokine response, Affect, Upper-body exercise, High-intensity interval training, Low-grade inflammation, Interleukin-6

## Abstract

**Purpose:**

To investigate the inflammatory and perceptual responses to three different forms of upper-body exercise.

**Methods:**

Twelve recreationally active, able-bodied males performed three work-matched arm-crank sessions in a randomised order: 30 min moderate-intensity continuous (CON), 30 min moderate-intensity with changes in cadence (CAD) and 20 min high-intensity interval training (HIIT). Blood samples were taken pre, post and 2-h post-exercise to determine plasma concentrations of interleukin (IL)-6 and IL-1ra. Perceptual responses pre, during and following the trials were assessed using the Feeling Scale, Felt Arousal Scale, Ratings of Perceived Exertion (RPE) and the Physical Activity Enjoyment Scale (PACES).

**Results:**

All trials were evenly effective in inducing an acute inflammatory response, indicated by similar increases in IL-6 after exercise and in IL-1ra at 2-h post exercise for all trials. More negative affect and higher RPE were reported *during* HIIT compared to CON and CAD, whereas PACES scores reported *after* exercise were higher for HIIT and CAD compared to CON.

**Conclusions:**

When matched for external work, there was no difference in the inflammatory response to HIIT compared to moderate-intensity upper-body exercise. Although HIIT was (perceived as) more strenuous and affective responses were more negative *during* this mode, the higher ratings of enjoyment for both HIIT and CAD reported *after* exercise suggest that the inclusion of variation enhances enjoyment in upper-body exercise. As the fashion in which upper-body exercise is performed does not seem to influence the inflammatory response, it might be advised to prescribe varied exercise to enhance its enjoyment.

## Introduction

It is widely recognised that regular exercise has protective effects against chronic low-grade inflammation-associated chronic diseases, such as type 2 diabetes mellitus (T2DM) and cardiovascular disease (CVD) (Warburton et al. 2006). One of the proposed reasons is its anti-inflammatory effect (Petersen and Pedersen 2005). An important role in this effect is attributed to interleukin (IL)-6, which is a pleiotropic cytokine with inflammatory properties, but is also suggested to be the initiator of the anti-inflammatory response after exercise. Namely, an acute bout of exercise upregulates levels of IL-6, followed by a longer lasting elevation of the anti-inflammatory cytokines IL-10 and IL-1ra (Petersen and Pedersen 2005). In turn, regular long-term exercise potentially downregulates resting levels of pro-inflammatory cytokines indicative of low-grade inflammation [e.g. IL-6, tumor necrosis factor alpha (TNF-α) and C-reactive protein (CRP)] (Beavers et al. 2010). For an acute bout of exercise the elevation of circulating IL-6 concentration and the resulting anti-inflammatory effect are dependent on both the intensity and duration of the session, with 126-fold increases in IL-6 seen after an ultra-running event (Nieman et al. 2005) and no increases seen after 30 min of moderate-intensity walking (Markovitch et al. 2008). Through an appreciation of the link between acute responses to exercise and its long-term effects, we may be able to develop strategies to augment the acute inflammatory response to exercise and enhance its potential to combat chronic low-grade inflammation.

One increasingly popular form of endurance exercise is high-intensity interval training (HIIT) (Muller 1953). Whereas the more traditional continuous moderate-intensity exercise is recommended to last at least 30 min (Haskell et al. 2007), this less time consuming form of exercise consists of short bursts of high-intensity efforts of over 80% of the peak oxygen uptake (V̇O_2_) interspersed with low-intensity, active recovery (Gibala and Little 2010). Typically, HIIT sessions take not more than 20 min and have been shown to be equally or even more effective in inducing cardiovascular adaptations (Gibala et al. 2012) and improving performance (Milanović et al. 2015) compared to moderate-intensity continuous exercise. Investigating the acute inflammatory response, Leggate et al. (2010) and Wadley et al. (2015) showed that the increase in IL-6 after HIIT was greater compared to moderate-intensity continuous exercise, although this finding has not consistently been shown in the literature (Cabral-Santos et al. 2015; Kaspar et al. 2016).

While the aforementioned studies where all conducted in cycling or running, no such study exists for upper-body exercise. The smaller muscle mass involved in this form of exercise might hamper the acute inflammatory response (Hirose et al. 2004; Bergfors et al. 2005), making the exploration of exercise modes that can augment this response even more relevant. Moreover, the relatively high prevalence of inactivity and chronic diseases in the population for which upper-body exercise is most suited (i.e. wheelchair users) further adds to this notion (Bauman and Spungen 2008). As a recent review pointed out, alternatives to traditional moderate-intensity continuous exercise like HIIT might be a promising way forward to improve health in persons with a spinal cord injury (Nightingale et al. 2017). Although bouts of moderate-intensity continuous upper-body exercise have previously been shown to be sufficient to provoke an acute inflammatory response (Umemoto et al. 2011; Paulson et al. 2015), this study will make a first step into the investigation of the acute inflammatory response to alternative forms of upper-body exercise (e.g. HIIT).

Despite the proposed health benefits of HIIT, these can only be achieved when engaging in this type of activity on a regular basis. The Hedonic theory states that people are more inclined to repeat behaviour that they find pleasant. Hence, it is suggested that the affective response to and enjoyment of exercise are important factors in exercise adherence (Ekkekakis et al. 2008). Studies into the affective response to single bouts of continuous exercise have shown a negative relationship between exercise intensity and affective responses during the activity, possibly making HIIT less suitable for health promotion. However, Bartlett et al. (2011) and Jung et al. (2014) showed that there might be different mechanism involved in the perceptual responses to HIIT, shown by higher ratings of enjoyment (reported *after* the exercise bout) for HIIT compared to moderate-intensity continuous exercise despite more negative affective responses *during* HIIT. Whether this phenomenon also exists for upper-body HIIT is not yet clear. Possible factors that could alter the perceptual responses to upper-body exercise are the unfamiliarity of the participants to the task, the more dominant role of peripheral fatigue (Paulson et al. 2013) and the altered substrate metabolism compared to lower-body exercise (Sawka 1986). Nevertheless, an initial study in persons with a spinal cord injury showed that the enjoyment of upper-body exercise might also be enhanced using HIIT (Astorino and Thum 2016).

To gain further knowledge in the potential of this relatively new form of upper-body exercise, this study investigates the acute inflammatory and perceptual responses to three different modalities of upper-body exercise, with a particular focus on HIIT as a possible alternative form of exercise to prevent or combat chronic low-grade inflammation. It is hypothesised that the exercise-induced increase in IL-6 and IL-1ra will be similar between modalities and that, despite more negative affective responses reported *during* exercise, the enjoyment as reported *after* exercise will be higher for HIIT compared to the moderate-intensity modalities.

## Methods

### Participants

Twelve recreationally active, able-bodied males volunteered for this study. All participants were recruited from a student population. After being informed about the study procedure, they signed an informed consent form at the start of the first visit. The study was approved by the Loughborough University ethical advisory committee, in accordance with the Declaration of Helsinki.

### Study design

Participants visited the laboratory on four occasions. All exercise tests were performed on a Lode Angio arm-crank ergometer (Lode, Groningen, The Netherlands). In the first visit, participants reported their physical activity levels using a bespoke questionnaire, and a graded incremental exercise test (GXT) to exhaustion was performed to assess peak exercise capacity and determine the lactate anaerobic threshold (LTan). The cytokine and perceptual responses to the following exercise modalities, which were matched for external work, were compared:

- Moderate-intensity continuous exercise (CON): 30-min arm-cranking at 80% of the power output (PO) at LTan, with a target cadence of 80 rpm.
- Moderate-intensity exercise with changes in cadence (CAD): 30 min arm-cranking at 80% of the PO at LTan, alternating high cadence (1 min at 110 rpm) with low cadence (1 min at 50 rpm).
- High-intensity interval training (HIIT): 20 min arm-cranking, alternating hard (1 min at 200% PO at the LTan) and easy (1 min at 40% of the PO at LTan), with a cadence of 80 rpm.

### Graded exercise test

Prior to the GXT, the height of the arm-crank and position of the chair were adjusted so that the arms never exceeded shoulder height and would never be fully extended during rotation. Heart rate (HR) was continuously measured using radio telemetry (Polar PE4000, Kempele, Finland), and V̇O_2_ was determined using Douglas bags, which were analysed with a Servomex (Servomex 1440, Servomex Ltd, Crowborough, UK). For blood lactate (BLa) determination, capillary blood was taken from the right earlobe and analysed using a Biosen C-line (EKF Industrie, Elektronik GmbH, Barleben, Germany).

After a 3 min warm-up, followed by 2 min rest, the GXT commenced at 5 W, followed by incremental stages of 15 W every 3 min. Cadence was held constant at 80 rpm. Oxygen uptake and BLa were assessed in the last minute of every incremental stage and final minute. The test was stopped at volitional exhaustion or when the participant could no longer maintain the requested cadence. The highest V̇O_2_ was taken as the peak value, whilst the highest 30 s rolling average HR was taken as HR peak. The LTan was determined using the Dmax method (Cheng et al. 1992).

### Main trials

Prior to all trials, participants refrained from exercise, caffeine, alcohol, whilst they standardised their diet in the 24 h before the trials using a food diary. All trials commenced between 11 am and 1 pm after a 2-h fast, with the specific starting times standardised within participants and at least 2 days between trials. Participants were assigned to the three trials in a randomised, counter-balanced order using a 3 × 3 Latin square design. The exercise started with a 2-min warm-up at 20 W, immediately followed by the main trial. During the trials, HR was measured continuously, and expired air was collected for 1 min during minute 3, 11, 19, and 27 (latter only for CON and CAD) for V̇O_2_ determination. Participants were allowed to drink water *ad libitum*.

### Blood analyses

Blood from an antecubital vein was drawn into a K_3_EDTA vacutainer pre-, directly post and 2 h after completion of the trial. Prior to the pre- and 2-h post-exercise sample, participants were seated for 10 min. Directly after collection, plasma was separated using a centrifuge spun at 400 g for 5 min and stored at −80 °C until analysis. Haematocrit was determined using a microlitre centrifuge (Mikro20, Andreas Hettich GmbH, Tuttlingen, Germany). Haemoglobin concentration was determined in duplicate with the cyanmethaemoglobin method using a spectrophotometer (CECIL CE1011, Cecil Instruments Ltd., Cambridge, UK). Haematocrit and haemoglobin concentrations were used to correct for changes in plasma volume from baseline according to the method postulated by Dill and Costill (1974). Interleukin-6 and IL-1ra concentrations were determined using an enzyme-linked immunosorbent assay (ELISA), purchased from R&D systems (Minneapolis, US). Samples were analysed in duplicate, with an average coefficients of variation of 8.5 and 7.8% for IL-6 and IL-1ra, respectively.

### Perceptual measures

The acute affective responses were reported using the Feeling Scale (FS) (Hardy and Rejeski 1989) and the Felt Arousal Scale (FAS) (Svebak and Murgatroyd 1985). A resting value was given prior to exercise and responses during exercise were reported from the 5th and 6th min with 6 min intervals, followed by directly post- and 20-min post-exercise. Furthermore, a FS score for the complete session was requested 20-min post-exercise [session-FS (sFS)]. Local, central and overall ratings of perceived exertion (RPE) (Borg et al. 1987) were reported during exercise for the same time points as the affective response, while a session-RPE (sRPE) was given 20-min post-exercise.

Participants filled out the Physical Activity Enjoyment Scale (PACES) (Kendzierski & DeCarlo 1991) and reported their enjoyment on a 20 cm Visual Analogue Scale (VAS) (Svensson 2000) (“Enjoyment”) 20-min post-exercise. On the same scale, participants reported how enjoyable they would find it to engage in this form of exercise for 2 to 3 times a week in the coming month if they had to rely on their upper body for exercise as result of an injury (“Expected enjoyment”).

After completion of all three main trials, the 20-min post-exercise enjoyment examination was extended with the following question: “Which of the three exercise modalities did you enjoy most? (“Preference”)”. Furthermore, participants rated their fondness for each of the modalities on a 1–9 Likert scale separately (“Fondness”). Lastly, participants were asked to write down their reasons for the reported “Preference”.

### Statistical analysis

Participant characteristics and outcome measures are given in means and standard deviations. When the assumption of normality was violated, identified by the Shapiro–Wilk test, data were log transformed before analysis. This had to be done for the IL-6 data. A Greenhouse-Geisser correction was applied when the assumption of sphericity was violated, which was tested with Mauchley’s sphericity test. Scale data were analysed with parametric tests, in accordance with Bishop & Herron (2015) who support the robustness of F-tests with regards to ordinal data, in line with data analysis in similar studies (Bartlett et al. 2011; Jung et al. 2014). A one-way repeated measures ANOVA was performed for BLa at the end of the trial, HR, V̇O_2_ (average over the complete trial) and for the scores on the PACES, enjoyment, anticipated enjoyment, FS, sFS and sRPE. A two-way repeated measures ANOVA was performed for the affective responses pre, post and 20-min post-exercise. For the perceptual responses during the sessions (FS, FAS and RPE) a regression analysis curve was fitted with the data of every time point during exercise. The slope of the regression curve, the regression coefficient (rc), for every individual was used as a measure of progression in the perceptual responses during exercise and a one-way repeated measures ANOVA was used to test for differences in the rc between modalities. A Chi^2^ test was used to test the distribution of “Preference” for goodness-of-fit. For all ANOVAs, post hoc Bonferroni corrected tests were used for further inspection when statistical significance was reached. Statistical significance was set at *p* < 0.05. The 22nd version of the statistical software package SPSS (SPSS inc, Chicago, IL) was used for all analyses.

## Results

Participant characteristics and the results of the GXT are shown in Table 1. All participants were able to complete the three main trials. Physiological outcome variables confirm that there was a significant difference in intensity between the three different exercise modes. Mean HR, V̇O_2_ and final BLa were higher in HIIT compared to CAD and CON. Only V̇O_2_ differed between the CON and CAD condition, with higher values during CAD (Table 2).

**Table 1:** Participants descriptives (*n* = 12) and main incremental exercise test results

Parameter	Mean (SD)
Age (years)	22.5 (3.3)
Body mass (kg)	76.0 (11.9)
Height (m)	1.80 (0.08)
Physical activity level (hours/week)	4.33 (2.04)
PO peak (W)	107 (24)
V̇O_2_ peak (L/min)	2.72 (0.69)
Relative V̇O_2_ peak (mL/kg/min)	35.5 (4.51)
HR peak (beats/min)	177 (17)
PO at LTan (W)	65 (15)

*POpeak*: peak power output, *V̇O*_*2*_ *peak* peak oxygen uptake, *HRpeak* peak heart rate, *PO at LTan* power output at the lactate anaerobic threshold

**Table 2:** Physiological outcomes and the perceptual responses during and 20 min after the three trials

Parameter	CON	CAD	HIIT
HR (bpm)	117 (15)	127 (17)	151^b^ (12)
BLa (mmol/L)	3.15 (1.11)	5.27 (4.54)	9.63^b^ (2.24)
V̇O_2_ (L/min)	1.43 (0.25)	1.76^a^ (0.36)	2.04^b^ (0.38)
V̇O_2_/V̇O_2_ peak (%)	53.5 (7.3)	65.6^a^ (9.6)	76.5^b^ (8.7)
rc FS	0.00 (0.06)	−0.02 (0.03)	−0.11^b^ (0.12)
rc FAS	0.01 (0.03)	0.01 (0.02)	0.05 (0.07)
rc RPE local	0.08 (0.05)	0.06 (0.06)	0.19^b^ (0.18)
rc RPE central	0.06 (0.05)	0.05 (0.07)	0.19^b^ (0.12)
rc RPE overall	0.06 (0.05)	0.05 (0.05)	0.18^b^ (0.14)
sRPE	10.8 (1.9)	11.3 (1.8)	14.8^b^ (2.3)
sFS	3.08 (1.08)	2.75 (1.22)	1.25^a^ (2.14)
PACES	78.8 (10.2)	92.4^a^ (8.7)	90.2^a^ (14.2)
Fondness	4.42 (1.89)	5.92 (2.15)	6.00 (1.28)
Enjoyment	12.1 (3.1)	11.7 (4.3)	14.2 (2.3)
Expected enjoyment	9.8 (4.1)	11.1 (3.9)	12.3 (3.7)
Preference	*n* = 2	*n* = 4	*n* = 6

*HR* heart rate, *BLa* final blood lactate concentration, *V̇O*_*2*_ oxygen uptake, *rc* regression coefficient, *FS* Feeling Scale, *FAS* Felt Arousal Scale, *RPE* ratings of perceived exertion, *sRPE* session ratings of perceived exertion, *sFS* session feeling scale, *PACES* physical activity enjoyment scale

^a^Significantly different from other trial(s)

^b^significantly different from CAD and CON

**Fig. 1:**
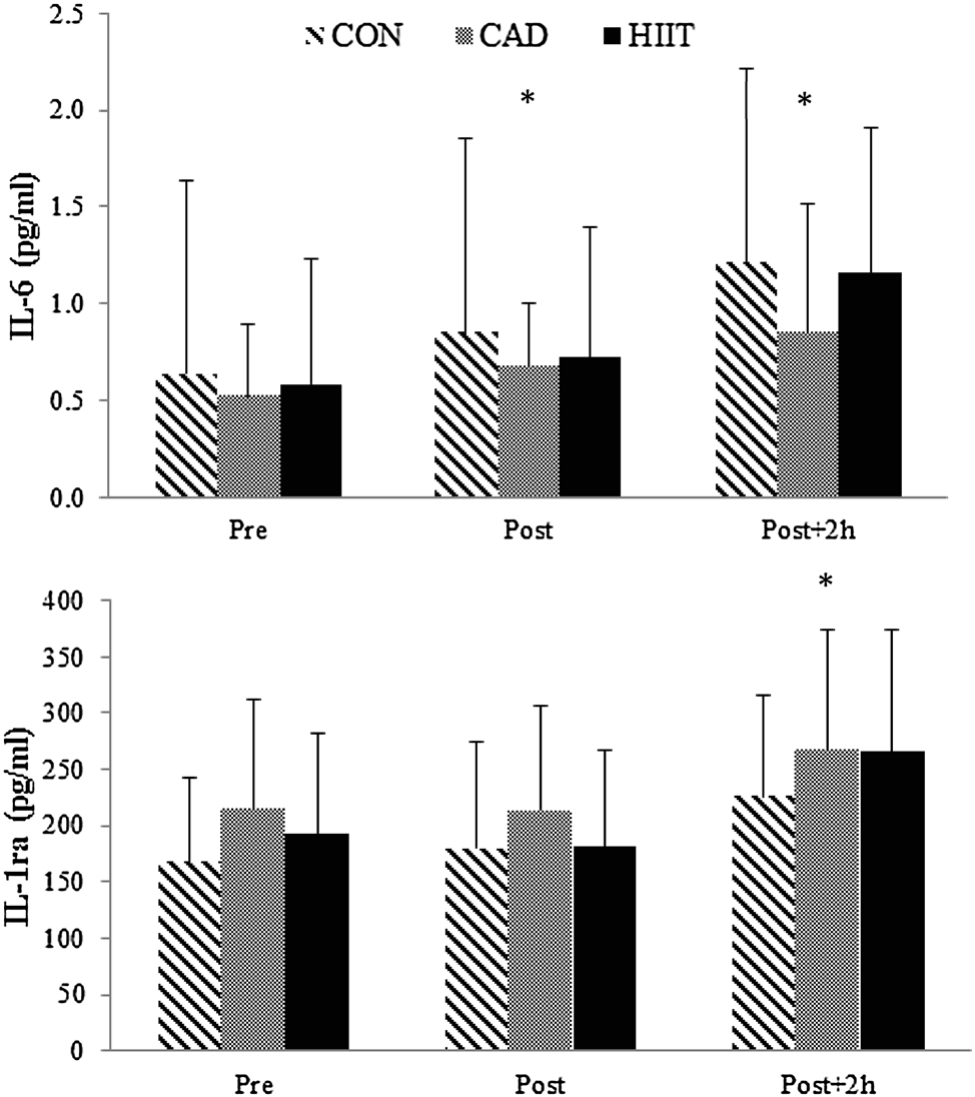
Plasma IL-6 and IL-1ra concentration pre, post and 2 h after the three upper-body exercise modalities. *CON* moderate-intensity continuous exercise, *CAD* moderate-intensity change in cadence, *HIIT* high-intensity interval training. *significant difference compared to previous time points

Both IL-6 and IL-1ra plasma concentrations increased significantly in response to the exercise bouts (Fig. 1). For IL-6 immediately post as well as 2-h post-exercise, values were higher compared to the preceding time point (*F*(1.14) = 22.4, *p* < 0.001), while levels of IL-1ra only increased at 2-h post-exercise (*F*(1.06) = 13.9, *p* = 0.003). The 95% confidence intervals (CI) for differences in IL-6 (in pg/ml) between pre and post-exercise were as follows: CON (0.03–0.39), CAD (0.03–0.20), HIIT (0.07–0.19), while the 95% CI for the differences between pre and 2-h post-exercise were: CON (0.11–1.04), CAD (0.29–0.57), HIIT (0.01–0.91). The 95% CI for differences in IL-1ra (in pg/ml) between pre and post-exercise were: CON (−129.4–80.3), CAD (−30.5–26.0), HIIT (−59.7–45.6); the 95% CI for the differences between pre and 2-h post-exercise were: CON (3.8–104.9), CAD (11.4–93.8), HIIT (2.4–139.6). No differences between the modalities or an interaction effect of “Time” × “Mode” was found for either of the cytokines (IL-6: *F*(2) = 1.86, *p* = 0.19; *F*(2.12) = 1.32, *p* = 0.29; IL-1ra: *F*(2) = 2.31, *p* = 0.35, *F*(1.59) = 0.68, *p* = 0.69).

**Fig. 2:**
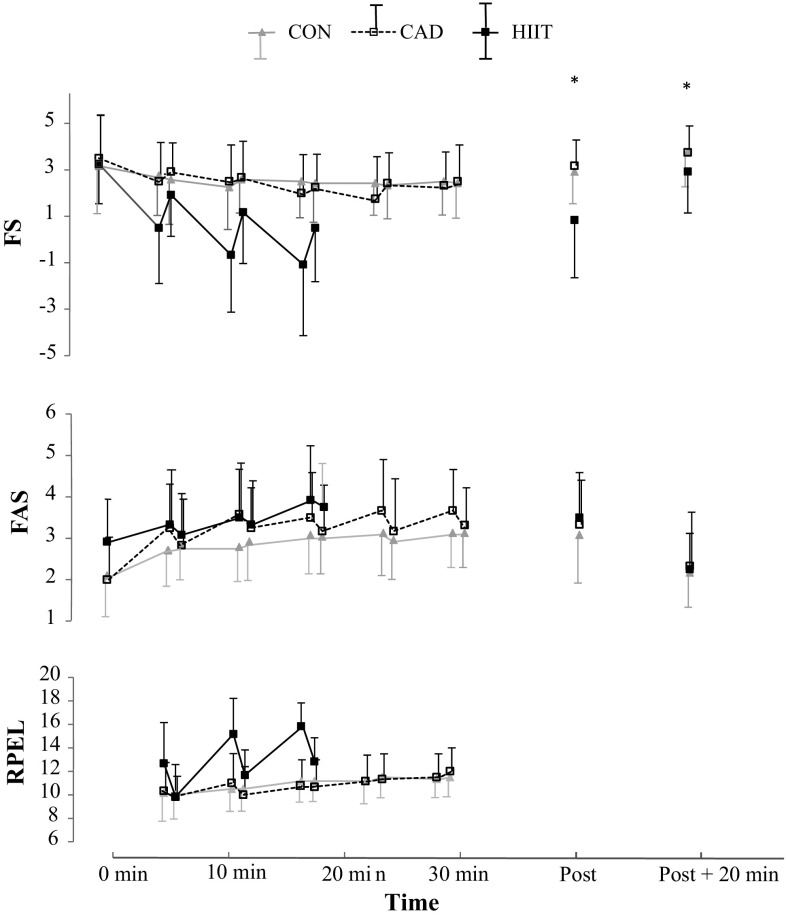
Perceptual responses pre, during and after the three different exercise bouts. *FS* Feeling Scale, *FAS* Felt Arousal Scale, *RPE L* local ratings of perceived exertion. * Denotes a significant difference between HIIT and the other modalities at that time point

The affective responses post and 20 min following the exercise bouts differed between the modalities. The FS scores showed significant effects for “Time” (*F*(2) = 4.12, *p* = 0.03), “Mode” (*F*(2) = 7.73, *p* = 0.003) as well as a time x mode interaction (*F*(2.15) = 4.79, *p* = 0.016). Scores on the FS in response to HIIT were significantly lower post and 20 min post-exercise compared to the other two modalities. The FAS scores were significantly increased directly post-exercise, without any differences between modalities (“Time” *F*(2) = 26.0, *p* < 0.001) (Fig. 2).

The progression of the affective responses during exercise, as assessed by the rc FS and the rc FAS, demonstrated a sharper decrease in scores on the FS during HIIT compared to the other modalities (*F*(2) = 7.65, *p* = 0.003, Table 2), indicating more negative affect during this exercise mode. There were no significant differences in the rc FAS between the modalities (*F*(1.33) = 2.97, *p* = 0.098). Of the three differentiated RPE scores reported during exercise, RPE C and RPE O showed a stronger increase during HIIT compared to CON and CAD (*F*(1.32) = 9.90, *p* = 0.004; *F*(1.10) = 6.75, *p* = 0.02, respectively), reflecting the demanding nature of the HIIT protocol. Detailed kinetics of the perceptual responses during the trials are shown in Fig. 2.

Although the sFS was significantly lower for HIIT compared to CON and CAD (*F*(2) = 8.09, *p* = 0.002) and the sRPE after HIIT was higher than after both other modalities (*F*(2) = 57.28, *p* < 0.001), participants rated both CAD and HIIT as more enjoyable than CON, as reported with the PACES (*F*(2) = 6.76, *p* = 0.005). In addition, a trend towards higher ratings of enjoyment for HIIT was reported with the VAS (*F*(1.26) = 3.36, *p* = 0.082). Although a similar trend was seen for fondness and expected enjoyment, neither of these two variables differed significantly between modalities (fondness: *F*(2) = 2.59, *p* = 0.098; expected enjoyment: *F*(2) = 2.55, *p* = 0.101). Although more participants reported to prefer HIIT and CAD compared to CON (Table 2), statistical significance was not reached (*p* = 0.37).

## Discussion

This study investigating the acute inflammatory and perceptual responses to three different arm-crank modalities showed a significant increase in plasma levels of IL-6 and IL-1ra after all three exercise modes, without differences between trials. Despite perceiving HIIT to be more strenuous and reporting more negative affect during this modality, upon completion of the exercise, both HIIT and CAD were reported as more enjoyable than CON. Since enjoyment is suggested to be important in exercise adherence (Ekkekakis et al. 2008), the effectiveness of including variation into exercise to enhance enjoyment is encouraging for exercise prescription in populations for which upper-body exercise can be a suitable alternative form of physical activity (e.g. wheelchair users, elderly or obese individuals).

### Cytokine response to upper-body exercise

The increase of both IL-6 and IL-1ra after CON, CAD, and HIIT adds further support to the anti-inflammatory potential of upper-body exercise. While initially it was suggested that the limited muscle mass involved in arm exercise might be insufficient to provoke an inflammatory response (Hirose et al. 2004; Bergfors et al. 2005), recent studies have shown the elevation in plasma concentration ofIL-6 and IL-1ra after upper-body exercise ranging from 30 min at moderate intensity (Paulson et al. 2015) to a wheelchair marathon (Sasaki et al. 2014). Moreover, Leicht et al. (2016) recently showed a similar acute inflammatory response to arm-compared to leg exercise when performed at the same relative intensity. Since the acute inflammatory response seems to be intensity and duration dependent, the initial lack of responses in IL-6 after upper-body exercise might have been caused by the short duration and the isolated muscle groups used for the exercise (Hirose et al. 2004; Bergfors et al. 2005). Indeed, as in running and cycling, the increases in IL-6 following upper-body exercise show a positive relationship with the physical demands of the exercise bout, with a ~1.8-fold increase found in the current study and almost 20-fold increases after a wheelchair marathon (Sasaki et al. 2014).

In that light, it might be conceived as somewhat surprising that the increase of IL-6 and IL-1ra did not differ between the three exercise modalities used in this study, despite higher RPE during HIIT. However, Fischer (2006) particularly highlights the importance of exercise duration in the elevation of IL-6 plasma concentration, which may be a reason why the shorter duration HIIT protocol did not induce a more pronounced inflammatory response when compared to the other modalities. Indeed, the present study suggests that the amount of “work” done is of greater importance than the fashion in which this work is prescribed. The execution of high-intensity bursts, with the accompanying rise in BLa, does not seem to have additional effects on the response of the cytokines measured in this study. This corroborates with studies examining the acute inflammatory response to different forms of cycling (Cabral-Santos et al. 2015), although larger increases in IL-6 after HIIT compared to work-matched moderate continuous exercise have been found as well (Leggate et al. 2010). Of note, as in the study of Leggate et al. (2010), the main trials of the current study were matched for external work rather than energy expenditure. This could be a possible limitation due to the possible influence of some aspects of the different trials on energy expenditure (e.g. the influence of cadence and intensity on arm-cranking mechanics and hence efficiency in individuals unaccustomed to upper-body exercise).

### Perceptual responses to upper-body exercise

While a relatively large body of literature exists on affective responses and ratings of enjoyment to different forms of lower-body endurance exercise (Bartlett et al. 2011; Jung et al. 2014; Kilpatrick et al. 2015), this is not the case for exercise performed with the upper-extremities. Insight in this modality could especially be useful for disabled, elderly or obese individuals, for which this form of exercise can be a suitable alternative to cycling or running. However, physiological as well as perceptual responses seen in cycling and running are not necessarily transferable to upper-body exercise. Differences in fibre-type composition, substrate metabolism and the larger role for peripheral fatigue during upper-body exercise could alter the perceptual responses compared to leg exercise (Sawka 1986). Moreover, arm-cranking is a task that most able-bodied and recently injured individuals are unaccustomed to, in contrast to cycling or running.

Nevertheless, in accordance with studies on lower-body exercise (Bartlett et al. 2011; Jung et al. 2014), in the current study, higher ratings of enjoyment were reported after completion of the intermittent exercise modes when compared to the continuous modality. Interestingly, this was despite higher RPE and more negative affective responses *during* HIIT. This seemingly contradictive phenomenon in intermittent exercise can be an important finding for future research into the optimisation of exercise adherence.

The Dual-Mode theory suggests that the affective response during exercise is intensity dependent, with variability between individuals in affective responses at intensities around the LTan, but an almost anonymous decline in affect during exercise intensities that surpass the LTan (Ekkekakis et al. 2008). For this reason, the intensity of the moderate intensity trials in the current study was set below this threshold. Our results support the Dual-Mode theory, with lowest scores on the FS during HIIT, the only modality were participants surpassed the LTan. In a previous study, using bouts of continuous lower-body exercise, the negative affective responses during exercise were also reflected in the ratings of enjoyment after exercise (Jung et al. 2014). However, the link between affective responses *during* exercise and *post*-exercise ratings of enjoyment might be different for intermittent exercise, shown by the current and other studies (Bartlett et al. 2011; Jung et al. 2014; Kilpatrick et al. 2015). For example, participants in the current study mentioned “a feeling of accomplishment” and “less boring/more interesting” as reasons why they reported to prefer HIIT over the other modalities (data not shown). This discrepancy between affect and enjoyment could be further explained by the difference in construct that the two measures embody. While the affective response is a hedonic, core response based on direct bodily sensations, enjoyment is regarded as an emotion, which is likely to require cognitive appraisal in addition. Ratings for enjoyment therefore might take into account factors such as the relevance of the session to achieve goals and the perceived ability of the participant to reach those goals (Martinez et al. 2015).

Nevertheless, the challenging character of HIIT is an often heard concern with regards to exercise prescription in non-athletic populations (Biddle and Batterham 2015). However, studies in individuals with T2DM (Maillard et al. 2016) and patients with heart disease (Currie et al. 2013) show promise for the prescription of HIIT with carefully chosen intensities to incorporate into regular exercise routines. In both long-term training studies, adherence rates did not differ between HIIT and moderate-intensity continuous exercise (Currie et al. 2013; Maillard et al. 2016). In addition, a recent study showed that HIIT is tolerated and perceived more enjoyable than continuous arm-crank exercise in a group of individuals with SCI (Astorino and Thum 2016). Together with the results of the current study, this suggests that also when performed with the upper body, intermittent and more challenging, but time efficient forms of exercise can be perceived as more enjoyable than continuous exercise. Notwithstanding, a novel finding of this study is that intermittent exercise without increases in intensity (which could enhance the “feelings of accomplishment”) can also enhance ratings of enjoyment, as shown by higher enjoyment for CAD compared to CON. This could be useful for individuals that are physically not yet ready to include HIIT in their exercise program or perceive the high intensities as aversive. Whether the phenomenon of higher ratings of enjoyment despite larger (feelings of) effort seen in HIIT can be used to better promote exercise cannot be concluded based on the data of the current (acute) study. Promising evidence includes data by Williams (2008) who showed that positive affective responses during exercise can predict increased physical activity levels 6 and 12 months later. For ratings of enjoyment and the comparison of different exercise modes, no prospective data on physical activity behaviour exist, neither for lower- nor upper-body exercise. In addition, future research on the health promoting potential of different modes of upper-body exercise should aim to include members of populations that can benefit most from upper-body exercise (i.e. wheelchair users), as it is not known how well the results of this study translate to these individuals.

## Conclusion

This study showed that three work-matched arm-crank modalities lasting 20–30 min can all induce an acute cytokine response. This response did not differ between CON, CAD and HIIT, suggesting that, if performed regularly, each modality could be equally effective in reducing chronic low-grade inflammation. This allows focus on factors that could enhance exercise adherence rates, notably the perceptual responses. While HIIT was (perceived as) more strenuous compared with CAD and CON, both intermittent modalities were rated as more enjoyable, suggesting that the inclusion of variation *per se* can enhance the enjoyment of exercise. Whether those enhanced ratings of enjoyment translate to higher adherence rates should be subject of future research.

## Abbreviations

ANOVA: Analysis of variance
BLa: Blood lactate
CAD: Change in cadence moderate intensity trial
CON: Continuous moderate intensity trial
CI: Confidence interval
CRP: C-reactive protein
CVD: Cardiovascular disease
Dmax: The maximal deviation method
(s)FS: (session) Feeling Scale
(s)FAS: (session) Felt Arousal Scale
GXT: Graded incremental exercise test
HR: Heart rate
HIIT: High-intensity interval training
IL: Interleukin
LTan: Lactate anaerobic threshold
PACES: Physical Activity Enjoyment Scale
PO: Power output
rc: Regression coefficient
(s)RPE: (session) Ratings of Perceived Exertion
T2DM: Type 2 diabetes mellitus
TNF-α: Tumor necrosis factor alpha
VAS: Visual Analogue Scale
V̇O_2_: Oxygen uptake
